# Heterologous activation of the *Hevea PEP16* promoter in the rubber-producing laticiferous tissues of *Taraxacum kok-saghyz*

**DOI:** 10.1038/s41598-020-67328-4

**Published:** 2020-07-02

**Authors:** Irisappan Ganesh, Sang Chul Choi, Sung Woo Bae, Jong-Chan Park, Stephen Beungtae Ryu

**Affiliations:** 1 Plant Systems Engineering Research Center, Korea Research Institute of Bioscience & Biotechnology (KRIBB), Daejeon, 34141 Republic of Korea; 2 Division of Bioengineering, KRIBB School, University of Science and Technology (UST), Daejeon, 34141 Republic of Korea; 3 Research & Development Center, DRB Holding Co. LTD, Busan, Republic of Korea; 4 Present Address: Department of Chemical Engineering, Pohang University of Science & Technology (POSTECH), Pohang, 37673 Republic of Korea

**Keywords:** Tissue engineering, Molecular engineering in plants

## Abstract

*Hevea brasiliensis*, the most abundant rubber crop, is used widely for the commercial production of natural rubber. To reduce the risk of a shortage in the supply of natural rubber that may arise from a single major rubber crop, rubber dandelion (*Taraxacum kok-saghyz*) has been developed as an alternative rubber-producing crop by using a transgenic approach. However, it is necessary to identify a suitable promoter for the transfer of rubber biosynthesis-related genes to the species. In this study, the promoter region of *H. brasiliensis PEP16,* which was isolated as a potentially important component in rubber biosynthesis, was sequenced and a p*PEP16::GUS* fusion construct was introduced into *T. kok-saghyz*. Histological and fluorometric studies using transgenic *T. kok-saghyz* plants indicated that the *HbPEP16* promoter was highly activated in a laticiferous tissue-specific manner under normal growth conditions and that promoter activation was tightly regulated by various hormones and external signals. These findings suggested that the *HbPEP16* promoter may be a useful molecular tool for the manipulation of gene expression in the laticiferous tissues of *T. kok-saghyz*.

## Introduction

Natural rubber (*cis*-1,4-polyisoprene) is an important raw material used in the manufacture of a wide variety of industrial products. Currently, it is sourced commercially from the Para rubber tree *Hevea brasiliensis*^1^, which produces an abundance of high-quality rubber that is relatively easy to harvest^2,3^. However, the decline in rubber tree plantations and the prevalence of life-threatening allergies to *Hevea* latex, coupled with an increase in demand, have stimulated research into the development of alternative rubber crops^4,5^. Although more than 2,000 plant species that produce natural rubber have been identified^6^, only a few of these species can produce comparable quantities of high-molecular-weight rubber to *Hevea*’s rubber^5,7^. Among these species, the Mexican shrub guayule (*Parthenium argentatum*) and the rubber dandelion (*Taraxacum kok-saghyz*) have been examined widely as alternative rubber yielding crops.

One strategy for the development of alternative rubber crops is the identification of the key regulatory genes in the rubber biosynthesis processes and manipulate the expression of these key genes in the laticiferous tissues where natural rubber is synthesized. To induce the expression of the target genes in the tissues, a laticiferous tissue-specific promoter is required. Although several studies have previously attempted to isolate laticiferous tissue-specific promoters^8–13^, a suitable promoter for the transformation of rubber dandelion *T. kok-saghyz* has not yet been identified.

HbPEP16 (GenBank no. MN326440) is a protein attached to the rubber particles in the latex of *H. brasiliensis*. In our rubber biosynthesis activity assays that examined the fractions of proteins dissociated from rubber particles, the fractions that showed enhanced rubber biosynthesis activities beyond basal levels commonly contained proteins such as HbPEP16, indicating that it may play a role in rubber biosynthesis processes^14^. The potential role of HbPEP16 in the rubber biosynthesis process is currently under investigation. This report has described the cloning of the *PEP16* promoter (GenBank no. MN200192) from *H. brasiliensis* and its heterologous expression in the latex of *T. kok-saghyz.* Histochemical GUS assays of transgenic *T. kok-saghyz* plants carrying the p*PEP16::GUS* construct showed that the *PEP16* promoter was highly activated under normal growth conditions in a laticiferous tissue-specific manner. Promoter activation was tightly regulated by hormones, such as abscisic acid (ABA), methyl jasmonic acid (MeJA), and auxin (NAA), and external signals, such as salt, light, and darkness. These findings indicated that *HbPEP16* was a suitable promoter for the laticiferous-specific expression of target genes in *T. kok-saghyz*.

## Materials and methods

### Plant materials and growth conditions

Rubber dandelion (*T. kok-saghyz*) seeds were obtained from USDA (accession number W6-35166) and multiplied to the third generation. The multiplied seeds were surface-sterilized in 70% ethanol for 30 s, immersed in 1% sodium hypochlorite for 20 min, and rinsed four times with sterile water^10^. The seeds were germinated on half-strength Murashige and Skoog (MS) medium with 3% (w/v) sucrose and 0.2% (w/v) phytagel in petri dishes under long-day conditions (16 h light with a light intensity of 45 µmol m^−2^ s^−1^ from 32 W cool white fluorescent tubes; 8 h of darkness at 25 °C for 1–2 months^10^. Plants were sub-cultured on fresh MS medium every 4 weeks. The plants were then transferred to soil pots in the greenhouse and grown for 2–3 months in preparation for sampling^10^.

### Isolation and sequence analysis of the *Hevea brasiliensis PEP16* promoter

**Figure 1:**
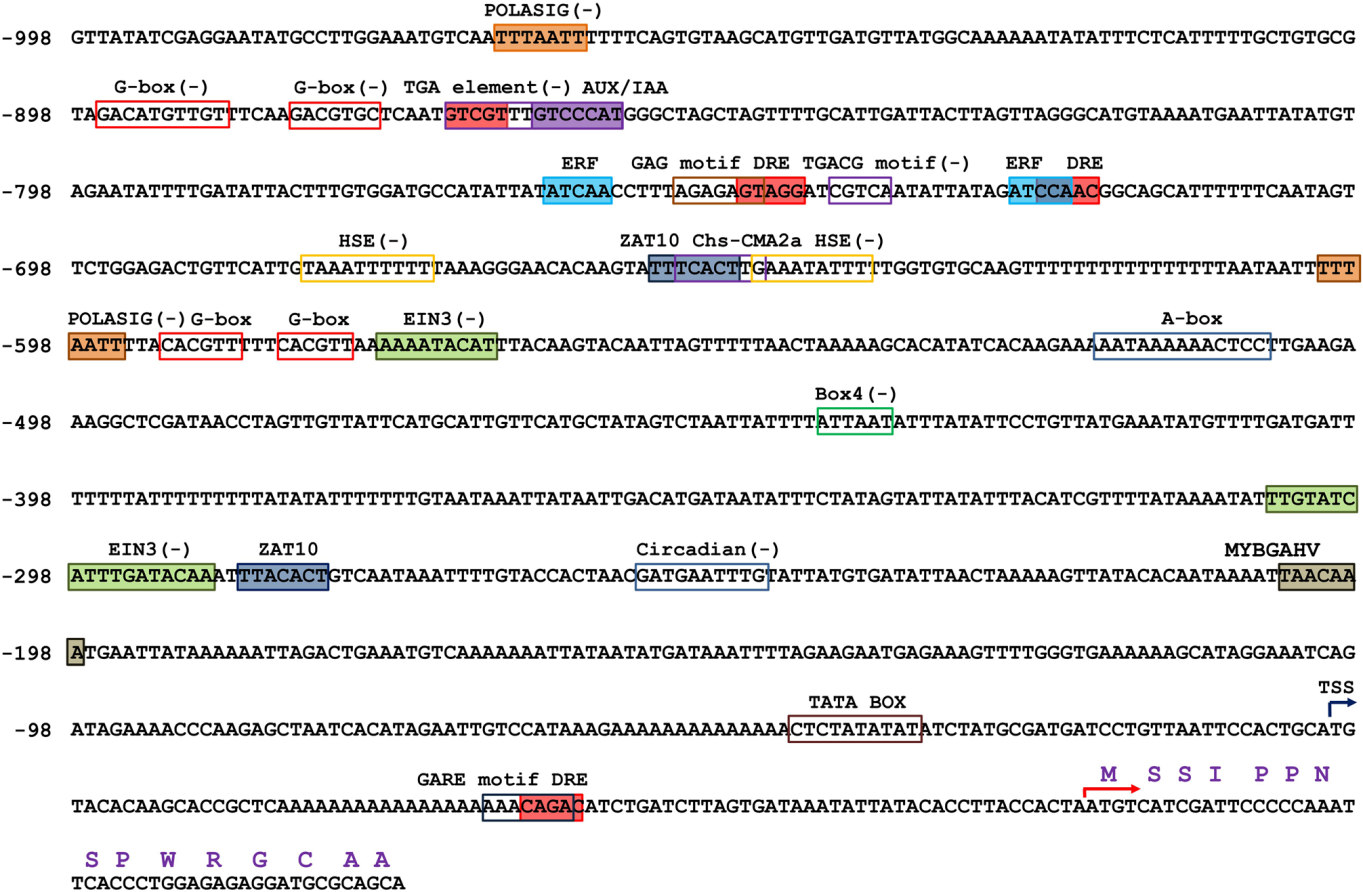
Nucleotide sequence of the *Hevea brasiliensis PEP16* promoter (GenBank no. MN200192) and putative *cis*-acting elements.

Genomic DNA (gDNA) was isolated from the young leaves of Para rubber trees, and gDNA extraction was performed following a previously described method^15^. The proximal promoter region of the *HbPEP16* gene was obtained by inverse PCR from the extracted gDNA^16^*.* The HincII*-*digested gDNA was self-ligated and used as a template for inverse PCR with primers designed from the first exon of the *PEP16* gene (Supplementary Fig. S1). The products for the first round of PCR were generated using a combination of primers (5′-GATGATATCGAATGCAGAAGC-3′ and 5′-CAAGACATCCTTCGCCATGT-3′; 5′-GATACTGCACCTTATCAACAC-3′ and 5′-CAGCATGGATTCGAAGCAAG-3′). The second round of PCR was performed using the 50 ×-diluted solution of the PCR products as a template, with the primers (5′-CTGAAAGTAATCAATCTGCAGC-3′ and 5′-GGATCACTATGTTCATCATAG-3′). Subsequently, the second-round PCR product was cloned into a pGEM-T vector (Promega) and sequenced (Supplementary Fig. S2). The sequence obtained was compared with previously published sequences in the NCBI database using BLASTN^17^. However, no significant sequence similarity was found. To identify *cis*-acting regulatory elements within the *PEP16* promoter, the promoter was analyzed using the PlantCARE database Fig. 1.

### Plasmid construction and transformation

**Figure 2:**
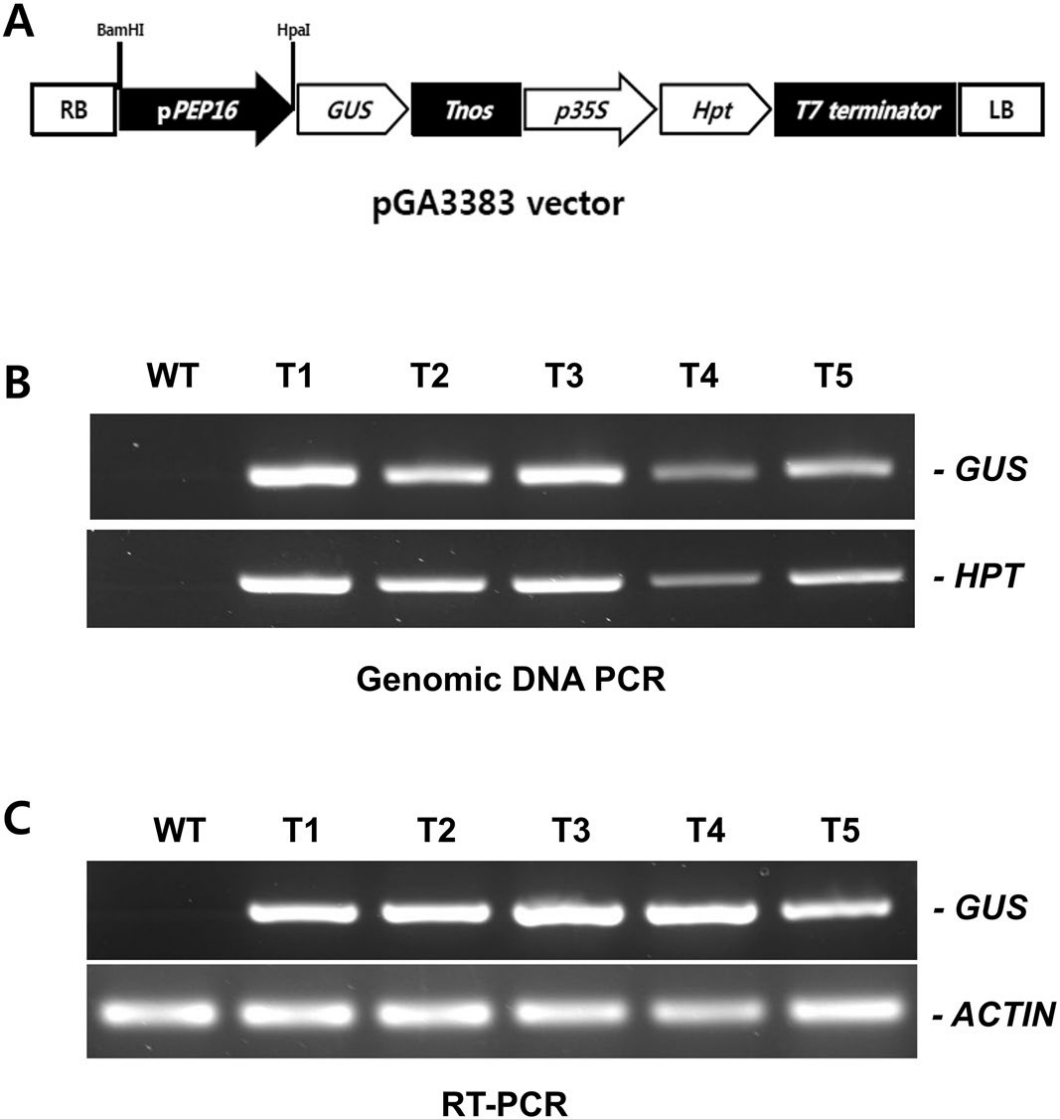
Transgenic *T. kok-saghyz* plants carrying the p*PEP16::GUS* construct. (**A**) A schematic diagram showing the structure of the modified pGA3383 vector that was used to construct p*PEP16::GUS*. (**B**) PCR amplification of the *HbPEP16* promoter and hygromycin gene (*HPT*) using genomic DNA of wild-type (WT) and transgenic dandelion lines (T1–T5) carrying the p*PEP16::GUS* construct with *HPT*. (**C**) RT-PCR analysis of *GUS* and *ACTIN* gene transcripts in wild-type (WT) and transgenic dandelion lines (T1–T5) carrying the p*PEP16::GUS* construct.

The pGEM-T cloned *PEP16* promoter (1079 bp) was subcloned into the pGA3383 binary vector^18^. The sequence was inserted between the *BamHI* and *HpaI* sites to generate the p*PEP16::GUS* fusion construct Fig. 2A. The p*PEP16::GUS* fusion construct was verified by sequencing. *Agrobacterium tumefaciens* LBA4404 was transformed with the construct using the freeze–thaw method^19^.

### Generation and identification of transgenic *T. kok-saghyz* plants

Transgenic *T. kok-saghyz* plants carrying p*PEP16::GUS* were obtained via *Agrobacterium*-mediated transformation^10,20^. Dandelion leaf explants were immersed in a suspension of *Agrobacterium* carrying p*PEP16::GUS* for 20 min at 25 °C and were shaken gently (50 rpm)^10^. The explants were co-cultured on regeneration medium [MS salts, 3% sucrose, 0.01 mg/L 3-indolebutyric acid (IBA), 2 mg/L benzyl aminopurine (BA), 0.3% phytagel, pH 5.8) for 3 days in the dark at 25 °C. After co-cultivation, the explants were washed with cefotaxime (500 mg/L) to remove *Agrobacterium*. The explants were then transferred to a regeneration medium supplemented with hygromycin B (25 mg/L) and cefotaxime (500 mg/L). After 3 weeks of culture in the dark, the tissues were maintained on the same medium for 4 weeks under continuous light conditions^10^. For rooting, explants with shoots were transferred to 1/2 MS medium containing 1.5% sucrose, 0.3% phytagel, and 25 mg/L hygromycin B at pH 5.8. In vitro rooted transgenic plants were transferred to small plastic trays containing top soil, vermiculite, and peat moss (2:1:0.5 v/v) and kept in a greenhouse.

### RT-PCR analysis

RT-PCR was performed to identify GUS expression in the root tissue of transgenic *T. kok-saghyz* plants carrying p*PEP16*::*GUS*. Total RNA was extracted from the root tissues using the Qiagen RNeasy Plant Mini Kit^21^. RNA samples were qualitatively and quantitatively analyzed using a NanoDrop-1000 spectrophotometer (Thermo Fisher Scientific, Wilmington, DE, USA). Approximately 1 µg of total RNA was reverse-transcribed using the TOP Script RT Dry Mix (Enzynomics, South Korea) according to the manufacturer’s protocol^22^. RT-PCR was performed with the prepared cDNA and GUS primers (5′-TGCAGATATTCGTAATTATGCG-3′ and 5′-CAACAGACGCGTGGTTACAG-3′)^10^. As a reference gene, the *T. kok-saghyz ACTIN* gene was amplified using the primers 5′-CTTTTCCATGTCGTCCCAGT-3′ and 5′-CTGGGTTTGCTGGTGATGAT-3′. The PCR products were visualized using ethidium bromide after separation on agarose gels^10^.

### Treatment of external signals and hormones

*In vitro*-*propagated* surviving plants were grown for 2 months in a greenhouse. The transgenic plants were then exposed to cold conditions, salt, dark conditions, light after dark conditions, and hormones. To induce salt stress, the plant roots were gently pulled out of the soil pots and dipped in 100 mM NaCl for 12 h. For cold and dark stresses, the plants were kept in the pots and placed in a cold room for 1 day (4 °C at 12 h/12 h day/night) and 5 days in darkness at 22 °C, respectively. After the 5-day dark period, the plants were kept under a light/dark (16 h/8 h) cycle of normal growth conditions for 12 h and 60 h. The roots of untreated plants grown under normal growth conditions were used as controls. For hormonal treatment, the plant roots were gently pulled out of the soil pots and dipped for 12 h in solutions of 100 µM naphthalene acetic acid (NAA), 100 µM gibberellic acid (GA_3_), 100 µM abscisic acid (ABA), 100 µM ethephon, and 100 µM methyl jasmonate (MeJA). Water was used as a control. The treated plants were harvested for histochemical and/or fluorometric GUS activity assays.

### Histochemical GUS activity analysis

The histochemical localization of GUS activity in transgenic *T. kok-saghyz* plants was performed using the substrate 5-bromo-4-chloro-3-indolyl-*β*-D-glucuronide (X-Gluc) with an oxidative catalyst^11,23^. The plant roots and leaves were washed with water and submerged in vials containing 1 × GUS solution (1 mM X-Gluc, 50 mM sodium phosphate buffer, 0.5 mM potassium ferrocyanide, 0.5 mM potassium ferricyanide, 10 mM EDTA, 0.1% Triton X-100, pH 7.0)^10^. The vials were incubated at 37 °C for 3 h in the dark. Pigments and chlorophyll were cleared by soaking the tissues in 95% ethanol. GUS staining in the longitudinal and transverse root sections was observed by using an anatomical microscope or Nikon Microphot-FXA microscope^10^. For the latex GUS assay, freshly tapped latex from the junction between the root and shoot was collected in ice-cold Eppendorf tubes containing 100 mM sodium phosphate buffer (pH 7.5)^10^. The collected latex was centrifuged (17,000 × *g*, 10 min, 4 °C) to separate the aqueous latex phase from the pellet^10^. Aqueous latex (60 µL) was collected and 6 µL of 10 × -concentrated GUS solution was added to a concentration of 1 × . The tubes were incubated in the dark in a 37 °C water bath for 3 h^10^.

### Fluorometric GUS activity analysis

Fluorometric assays of GUS activity were conducted following the method of Jefferson *et al.*^23^, to quantify the levels of GUS enzyme activity in the root tissues of transgenic *T. kok-saghyz* plants carrying p*PEP16*::*GUS*. Plant tissues were homogenized in a GUS assay buffer (50 mM potassium phosphate, 10 mM EDTA, 0.1% Triton X-100, 0.1% Sarcosyl, 2 mM DTT, and 10 μg/mL cycloheximide), and an aliquot of the supernatant was incubated after 4-methylumbelliferyl-*β*-D-glucuronide (4-MUG) was added as the substrate at 37 °C for 2 h^10^. The amount of 4-methylumbelliferone (4-MU) formed by the GUS reaction was determined using a 96-well microtiter plate reader. Protein concentrations were determined following the method described by Bradford^24^, using a Coomassie protein assay kit (Bio-Rad) with BSA as the standard.

### Measurement of transient chlorophyll *a* fluorescence

Chlorophyll fluorescence was measured from the longest leaves at the apex, middle, and base regions using the HANDY PEA fluorimeter (Hansatech, UK). Before all measurements, leaves were dark-adapted for 20 min. The light intensity was 3,500 μmol photons m^−2^ s^−1^, provided by an array of three high-intensity light-emitting diodes, focused on a spot of 5 mm in diameter, and recorded for 1 s with 12-bit resolution. Twelve readings per line were averaged using the HANDY PEA software (version 1.31). The chlorophyll *a* fluorescence (Fv/Fm) value was measured and analyzed according to the equations of the JIP test^25^.

### Statistical analysis

All data are expressed as the mean value of at least three biological replicates. The data were analyzed using *t*-tests, and differences were considered statistically significant for a *P* value of < 0.05^22^.

## Results

### *cis*-Regulatory element content of the *HbPEP16* promoter

The PlantCARE^26^ and PlantPAN 2.0^27^ databases were used to identify matches in the *HbPEP16* promoter to the *cis*-regulatory elements of other plant species Fig. 1. Several types of regulatory elements were identified in the *HbPEP16* promoter. These included *cis*-acting elements, Box 4, chs-CMA2a, G-box, GAG that are involved in responses to light, HSE which is involved in heat stress responsiveness; ERF, consisting of TC-rich repeat elements, which is involved in the defense response; and DRE and ZAT10, which function under drought, low temperature, and high salt tolerance. Also identified were circadian elements involved in the circadian control and *cis*-acting elements involved in phytohormone responsiveness including the GARE-motif for responding to gibberellin, EIN3 for responding to ethylene, the TGA-element for responding to auxin, and the TGACG motif involved in the response to methyl jasmonate. A-box, which is responsive to *α-*amylase promoters, was identified in the promoter region. These *cis*-acting elements may modulate gene expression in a tissue-specific manner during growth and development.

### Generation of transgenic plants containing the p*PEP16::GUS* construct

The main purpose of the current study was to isolate a promoter that was specifically activated in laticiferous tissues, to provide a tool to improve the quantity and quality of rubber in rubber-producing plants by engineering the rubber biosynthetic pathways more precisely. The rubber dandelion plant, *T. kok-saghyz,* is a good plant model as well as an alternative rubber crop owing to its ease of planting and short maturation time. Thus, transgenic *T. kok-saghyz* plants were generated to express p*PEP16::GUS*. Using inverse PCR, 1,079 bp of the *HbPEP16* promoter was isolated (Fig. S1) and sequenced (Fig. S2). The *HbPEP16* promoter fragment was cloned upstream of the *GUS* gene of the pGA3383 vector Fig. 2A. *T. kok-saghyz* plants carrying the p*PEP16::GUS* construct were generated using *Agrobacterium*-mediated transformation. The *T. kok-saghyz* transgenic plants carrying the p*PEP16::GUS* construct were verified using PCR analysis with *GUS* and *HPT* primers Fig. 2B.

### Basal expression analysis in p*PEP16::GUS* transgenic *T. kok-saghyz* plants

**Figure 3:**
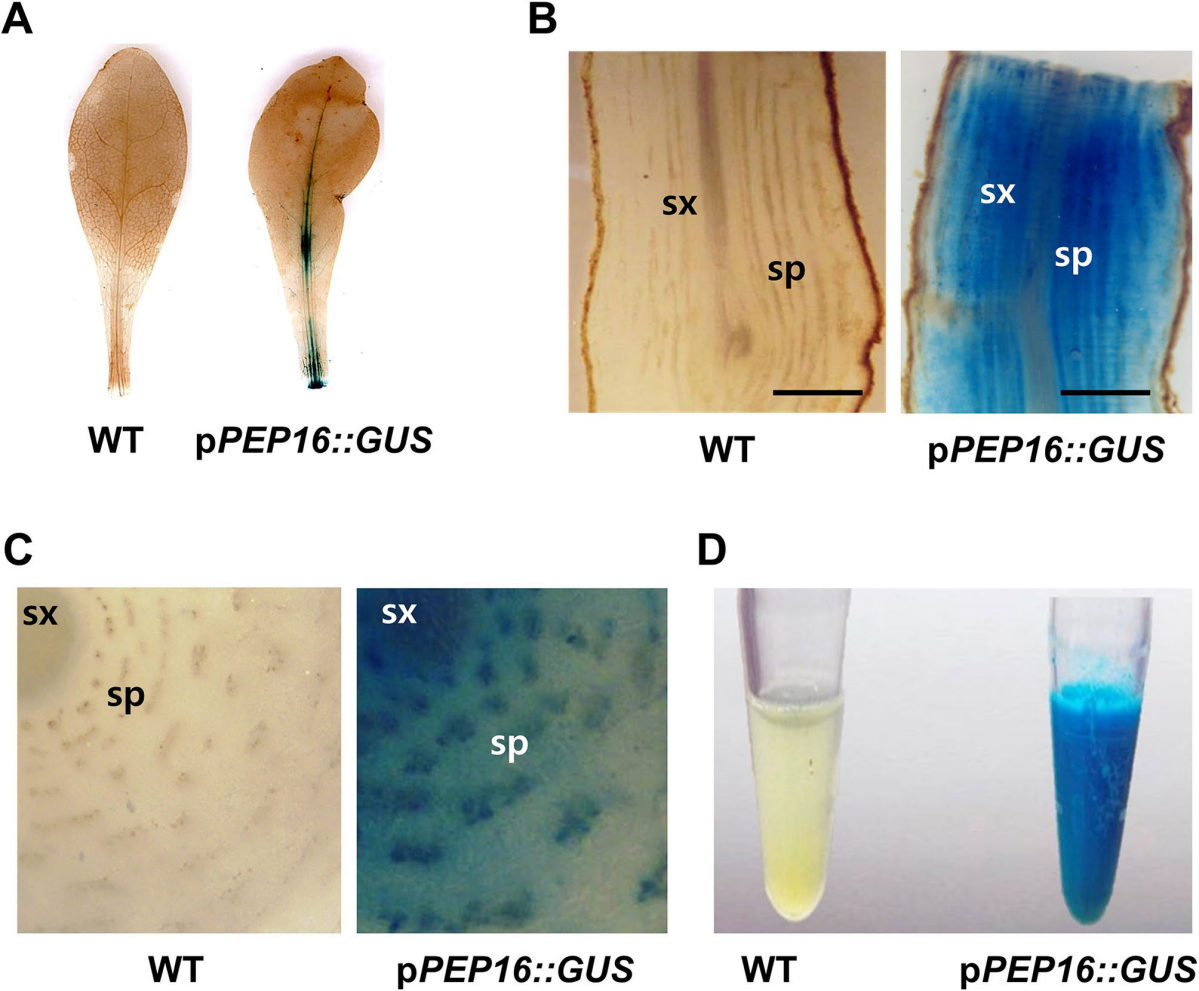
Laticiferous tissue-specific expression of *GUS* driven by the *HbPEP16* gene promoter in transgenic *T. kok-saghyz* plants. GUS stained images are shown for leaf tissues (**A**), longitudinally sectioned roots (**B**), transversely sectioned roots (**C**), and latex (**D**) of wild-type (WT) and transgenic plants carrying the p*PEP16::GUS*. Scale bar = 1 mm. sx, secondary xylem; sp, secondary phloem.

RT-PCR analysis showed that *GUS* expression was detected in the root sections of all five transgenic lines Fig. 2C. To examine the tissue-specific activation of the *HbPEP16* promoter, histochemical GUS staining was performed on the leaf tissues and longitudinally and transversely sectioned root tissues. Some GUS expression was detected in the main vein, but not in other parts of the leaf tissue of the transgenic *T. kok-saghyz* plants Fig. 3A. High GUS expression was observed in both the secondary xylem and phloem regions of both the longitudinally Fig. 3B and transversely Fig. 3C sectioned root tissues of the transgenic plants. Strong GUS staining was also observed in the latex solution obtained from the transgenic plants Fig. 3D. In contrast, Gus expression was not detected in the wild-type (WT) control Fig. 3A–D.

### Alteration of *HbPEP16* promoter activity by environmental signals

**Figure 4:**
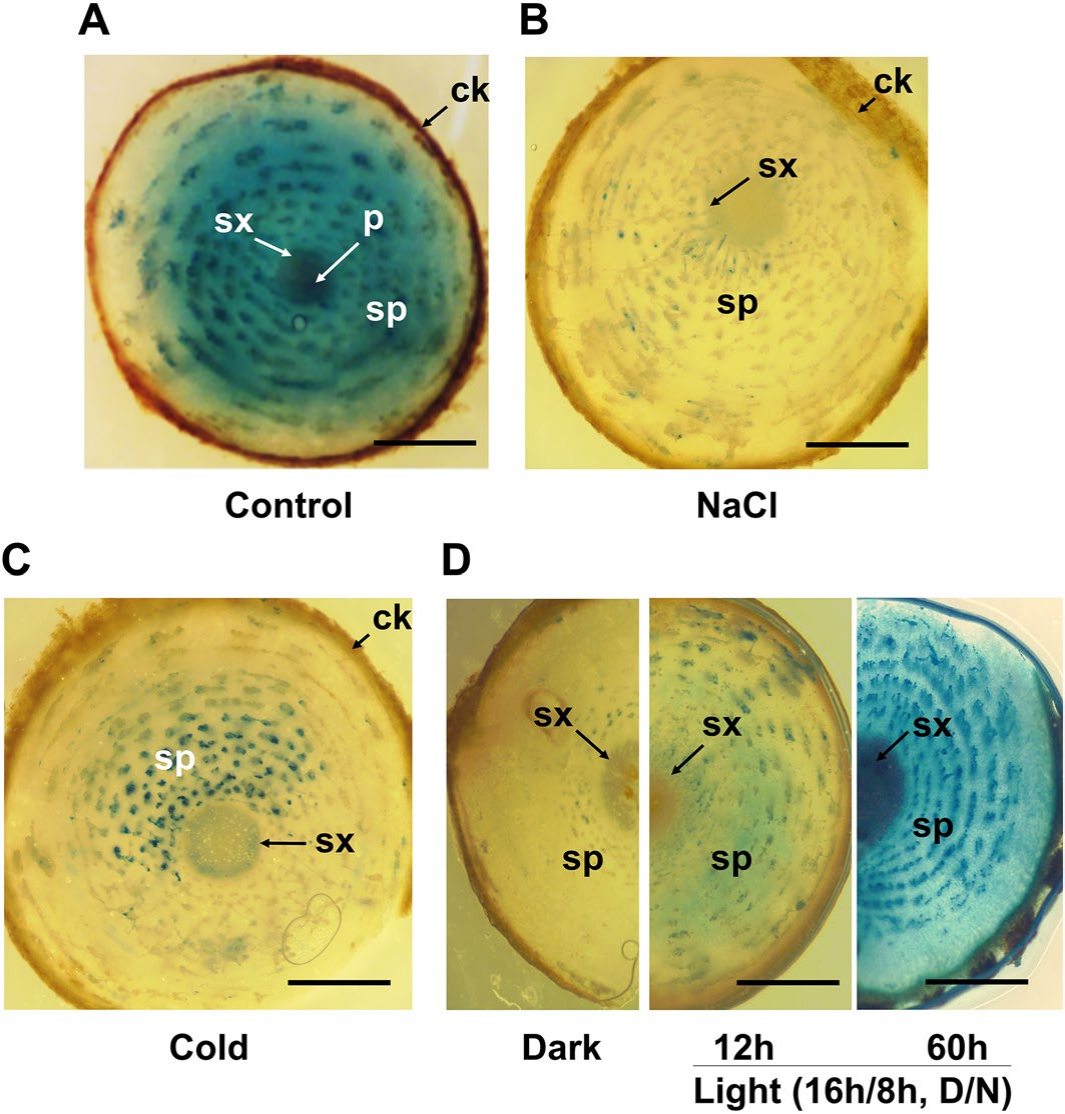
Regulation of the *HbPEP16* promoter by external signals such as salt, cold, dark, and light in transgenic *T. kok-saghyz* plants. GUS-stained images are shown for the untreated control under normal growth conditions (**A**), salt-treated (**B**), cold-treated (**C**), and 5 day-dark treatment (left), followed by 12 h (middle) and 60 h (right) normal growth light treatments (**D**) of transgenic plants carrying the p*PEP16::GUS* construct. Control and treated plants were kept in a 16 h photoperiod regime and the roots were transversely sectioned. Scale bar = 1 mm. sx, secondary xylem; sp, secondary phloem; ck, cork; p, pith.

**Figure 5:**
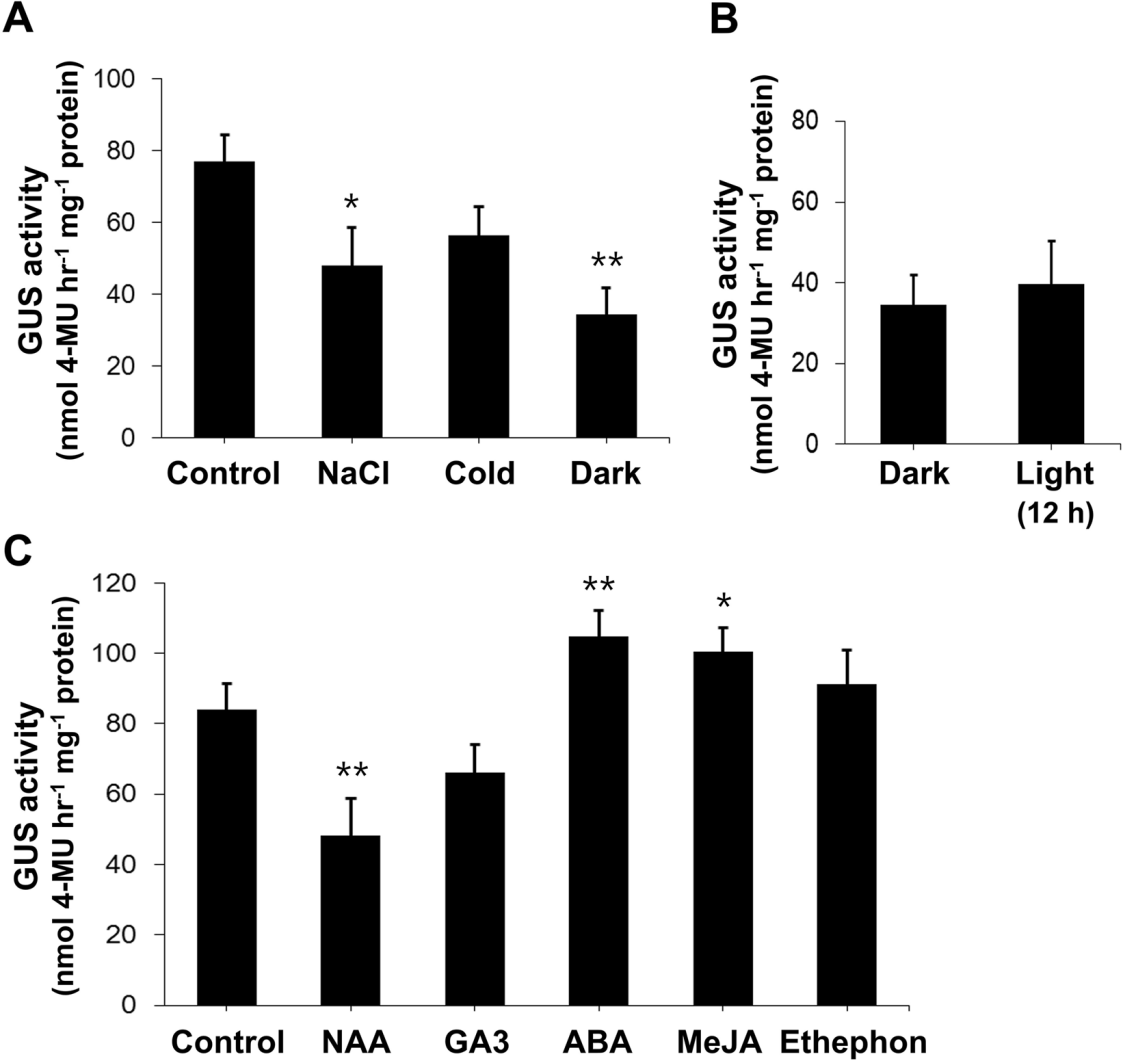
Fluorometric GUS activity assay of transgenic *T. kok-saghyz* plants in response to external and hormonal signals. The effects of external signals such as salt, cold, and dark stresses (**A**), dark stress, and 12 h light treatment after dark stress (**B**), and hormones such as auxin (NAA), gibberellin (GA_3_), ABA, MeJA, and ethylene-releasing ethephon (**C**) on fluorometric GUS activities driven by the *HbPEP16* promoter in transgenic *T. kok-saghyz* carrying the p*PEP16::GUS* construct*.* The asterisks indicate statistically significant differences (treatments versus control) as determined by *t*-tests: *P < 0.05, **P < 0.01.

Analysis of the *HbPEP16* promoter sequence revealed a few *cis*-elements for light, low temperature, and salt-based signals. To test this possibility, the *HbPEP16* promoter activity in transgenic *T. kok-saghyz* plants carrying p*PEP16::GUS* was investigated in response to dark, light, salt, and cold conditions. Histochemical staining showed that the *HbPEP16* promoter as highly activated in the laticiferous tissues of untreated control transgenic plants under normal growth conditions Fig. 4A. However, when exposed to the salt (100 mM NaCl) treatment, GUS expression was dramatically lower in the laticiferous tissues of the treated plants than in those of control plants under normal growth conditions Fig. 4B. The fluorometric assay revealed that the quantitative results of GUS activity were consistent with those of histochemical studies Fig. 5A. In response to the cold treatment, GUS expression was also lower than that in the control Fig. 4C. The fluorometric assay showed lower GUS activity under cold treatment, but this was not significant Fig. 5A. GUS expression was greatly reduced following the 5-day dark condition Fig. 4D, left), but resumed slightly at 12 h Fig. 4D, middle) and completely at 60 h Fig. 4D, right) after the plants were returned to normal growth light conditions (16 h/8 h, D/N). The fluorometric assay also revealed similar patterns: a dramatic de-activation of GUS activity under the dark treatment, a slight, prompt reactivation 12 h after being returned to the light treatment Fig. 5B^,^ and full reactivation of GUS activity to the levels of the untreated control Fig. 5A at 60 h after transfer to the normal growth light conditions (data not shown). GUS expression was primarily observed as a dot-like shape that formed concentric circles in the transverse section of root tissues.

### Regulation of the *HbPEP16* promoter by hormonal signals

Analysis of the *PEP16* promoter sequence revealed *cis*-elements for hormones such as ethylene, auxin, GA_3_, MeJA, and ABA (drought). To evaluate the hormonal regulation activity of the *HbPEP16* promoter in transgenic *T. kok-saghyz* plants carrying p*PEP16::GUS,* GUS activity was measured after the roots of transgenic plants were treated with the different hormones. By fluorometric analysis, the highest GUS activity was measured in the ABA treatment Fig. 5C. MeJA enhanced GUS activity to levels comparable with those achieved by ABA. A significant reduction in GUS activity was observed after auxin (NAA) treatment. However, treatments with GA3 and ethylene-releasing ethephon did not affect GUS expression significantly, although a slight decrease or increase in the activity was observed, respectively Fig. 5C.

## Discussion

Owing to the increased demand for natural rubber, and the risk of collapse of the current major rubber crop, *Hevea*, an alternative source of rubber is required. *T. kok-saghyz* is grown widely in various temperate zones and may be a good alternative source of natural rubber, as the rubber quality of *T. kok-saghyz* is similar to that of *H. brasiliensis*^1^. To be competitive as a commercial rubber crop, *T. kok-saghyz* needs to be more productive and have a higher rubber content. To enhance the rubber yield in *T. kok-saghyz*, transgenic approaches have been studied^1^. To express the transferred genes in the laticiferous tissues of *T. kok-saghyz,* where rubber biosynthesis occurs, molecular tools, such as tissue-specific promoters, are required. In a previous study with *Taraxacum brevicorniculatum*^10^, we reported a laticifer tissue-specific promoter, the *Hevea SRPP* gene promoter. However, the *HbSRPP* promoter activity is only weakly detected under normal growth conditions and strongly activated under cold conditions, which are not favorable for rubber biosynthesis. In addition, the *ToPPO-1* promoter also showed weak activity in the GUS staining of transgenic dandelion plants grown at normal temperatures^11^. In this study, we isolated the promoter of the *HbPEP16* gene, which is strongly activated under normal growth conditions but inactivated under stress conditions, including salt and darkness. Whether the expression levels are desirable or sufficient may be dependent on target genes, as the required expression level differs according to their cellular functions. It is also important to consider whether the promoter activity is modulated in parallel with the activity of the specific target metabolism, for example, rubber biosynthesis.

The transgenic *T. kok-saghyz* plants were generated to carry p*PEP16::GUS* because *HbPEP16* was identified in our preliminary studies as a potential component of the rubber biosynthesis process. Strong GUS activity driven by the *HbPEP16* promoter was observed in root sections of the transgenic plants. The *HbPEP16* promoter activity was observed in dot-like concentric ring structures in the secondary phloem regions of the transversely sectioned *T. kok-saghyz* roots. Strong GUS activity driven by the *HbPEP16* promoter was also pronounced in the latex of *T. kok-saghyz*. In contrast, the control (WT) plants showed no GUS activity and a low level of GUS staining was detected only in the main vein of the leaf tissue of the transgenic plants, where some latex was also present. This clearly showed the expression of the *HbPEP16* promoter in the laticiferous tissues of *T. kok-saghyz*. Notably, the transgenic *T. kok-saghyz* plants showed no abnormality in photosynthetic efficiency compared with the WT. The values of F0, Fm and Fv/Fm were not significantly different between WT and the *T. kok-saghyz* transgenic plants (Supplementary Fig. S3).

The *cis*-acting elements responding to abiotic stresses such as light, salt, and cold were predicted in the *HbPEP16* promoter sequence. Therefore, it was expected that the *HbPEP16* promoter was regulated by these external signals. The histochemical data indicated that the *HbPEP16* promoter was greatly downregulated in response to external signals such as salt and dark stresses. The fluorometric assay showed quantitative results that were consistent with those obtained with the histochemical GUS expression assay. In contrast, the light signal was found to positively regulate the *HbPEP16* promoter immediately in plants subjected to darkness. This may be due to the presence of multiple *cis*-acting elements, namely Box-4, chs-CMA2a, G-box, and GAG motif, which are responsive to light.

In addition to abiotic stress regulatory elements, *cis* elements responding to hormones such as ABA, ethephon, MeJA, GA_3_, and NAA were also identified in the promoter region of *HbPEP16*. ABA responds to various environmental stressors^28,29^. Following ABA treatment of the root tissues of the transgenic *T. kok-saghyz* plants, GUS expression was enhanced in the root laticiferous tissues compared with the control, indicating that the promoter activity was enhanced by the ABA hormone. MeJA plays an important role in plant defense reactions^30^. Upon treatment of the transgenic *T. kok-saghyz* plant root tissues with MeJA, increased GUS expression was observed in the root tissues, to a similar extent as that observed with ABA treatment. Previous studies have shown that exposure to exogenous MeJA and wounding can stimulate natural rubber production in rubber trees^31,32^. Furthermore, JA is believed to be a general inducer of natural rubber biosynthesis and activator of some genes related to it^33,34^. Therefore, these results support the hypothesis that the *HbPEP16* promoter is activated in a synchronized manner during rubber biosynthesis activity. Ethylene, a ripening hormone, plays an important role in the defense mechanism against pathogen attack/invasion. Ethephon treatment of the root tissues did not significantly affect the promoter activity. It was previously reported that ethylene stimulated latex production, but had little direct effect on the acceleration of rubber biosynthesis in *H. brasiliensis* when applied as ethephon^35^. The phytohormone gibberellin (GA_3_) is a significant growth regulator in promoting plant growth^36^ and the antagonistic interactions between ABA and GA responsive elements have been reported^37,38^. Auxin, another plant growth-promoting hormone, downregulated the promoter activity to a greater extent*.* In line with this, the exogenous auxin treatment of plant root tissues has been found to inhibit primary root growth^39^.

Both external and internal signals influenced *HbPEP16* gene promoter activity. In rubber plants, rubber is produced from the stored photosynthetic products^1^ and it has been proposed that rubber content per latex volume varies diurnally owing to the diurnal variation in HMG-CoA reductase activity^40^. The reduced *HbPEP16* activity under prolonged darkness observed in this study may be due to the absence or reduction in the major photosynthetic components involved in rubber biosynthesis, namely acetyl-CoA, NADPH, and ATP. Decreased GUS expression under salt or dark stress conditions denotes the downregulation of the *HbPEP16* promoter in unfavorable environments. Among plant hormones, *cis* elements responding to ABA and MeJA positively affected the *HbPEP16* promoter when the root tissues were directly exposed to the hormones. It should be noted that the effects of the hormones on *HbPEP16* promoter activity in the root tissues may be different when the hormones are treated on the leaf tissues. This remains to be further investigated in the future.

In conclusion, we identified and characterized the *HbPEP16* gene promoter, which can drive tissue-specific gene expression during the growth and development of *T. kok-saghyz* plants. This study demonstrates that *HbPEP16* is a laticiferous tissue-specific promoter, which is highly activated under normal growth conditions and is tightly regulated by several internal and external stimuli. This may facilitate the appropriate spatial and temporal expression of transfer genes in transgenic plants of the rubber-producing dandelion.

## Supplementary information

Supplementary file1
