# Supplementary material for: Heterologous activation of the Hevea PEP16 promoter in the rubber-producing laticiferous tissues of Taraxacum kok-saghyz
Source: Sci Rep. 2020 Jul 2;10:10844. doi: 10.1038/s41598-020-67328-4 (PMC7331677; doi:10.1038/s41598-020-67328-4)
Supplement: Supplementary file 1 — Supplementary file1 [file 41598_2020_67328_MOESM1_ESM.pdf]

Supplementary Information

Heterologous activation of the *Hevea PEP16* promoter in the rubber producing laticiferous tissues of *Taraxacum kok-saghyz*

Irisappan Ganesh[1,3,#], Sang Chul Choi[1], Sung Woo Bae[1,2], Jong-Chan Park[1] and Stephen Beungtae Ryu[1,2,*]

[1]Plant Systems Engineering Research Center, Korea Research Institute of Bioscience & Biotechnology (KRIBB), Daejeon 34141, Republic of Korea.

[2]Division of Biosystems and Bioengineering, University of Science and Technology (UST), Daejeon 34141, Republic of Korea.

[3]Research & Development Center, DRB Holding Co. LTD, Busan, Republic of Korea.

[#]Current address: Department of Chemical Engineering, Pohang University of Science & Technology (POSTECH), Pohang 37673, Republic of Korea.

*Corresponding author

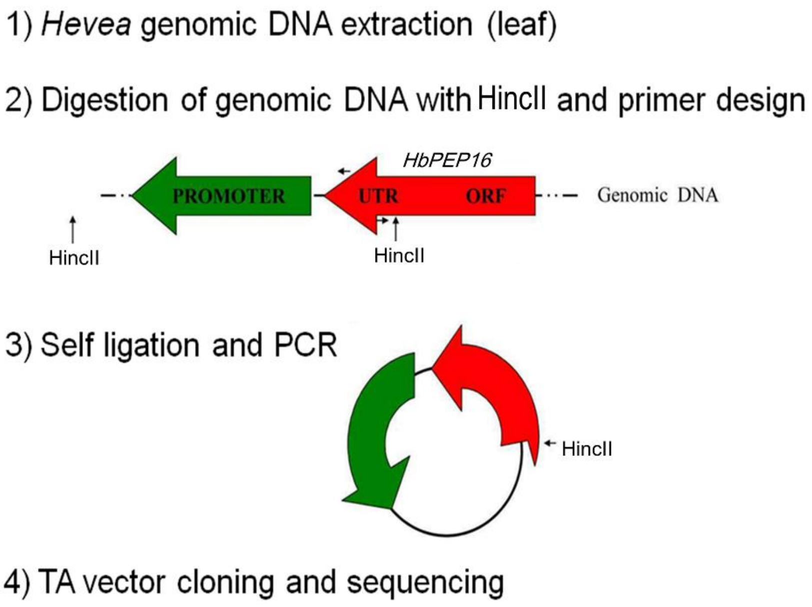

Supplementary Figure S1. Schematic representation of the methodology for isolation of *Hevea PEP16* promoter.

GTTATATCGAGGAATATGCCTTGGAAATGTCAATTTAATTTTTTCAGTGTAAGCATGTTGATGTTATGGCAAAAAATATATTTCTC
ATTTTTGCTGTGCGTAGACATGTTGTTTCAAGACGTGCTCAATGTCGTTTGTCCCATGGGCTAGCTAGTTTTGCATTGATTAC
TTAGTTAGGGCATGTAAAATGAATTATATGTAGAATATTTTGATATTACTTTGTGGATGCCATATTATATCAACCTTTAGAGAGTAG
GATCGTCAATATTATAGATCCAACGGCAGCATTTTTTCAATAGTTCTGGAGACTGTTCATTGTAAATTTTTTTAAAGGGAACACA
AGTATTTCACTTGAAATATTTTTGGTGTGCAAGTTTTTTTTTTTTTTTTTAATAATTTTTAATTTTACACGTTTTTCACGTTAAAAAAT
ACATTTACAAGTACAATTAGTTTTTAACTAAAAAGCACATATCACAAGAAAAATAAAAAACTCCTTGAAGAAAGGCTCGATAACC
TAGTTGTTATTCATGCATTGTTCATGCTATAGTCTAATTATTTTATTAATATTTATATTCCTGTTATGAAATATGTTTTGATGATTTTT
TTATTTTTTTTTATATATTTTTTTGTAATAAATTATAATTGACATGATAATATTTCTATAGTATTATATTTACATCGTTTTATAAAATATTT
GTATCATTTGATACAAATTTACACTGTCAATAAATTTTGTACCACTAACGATGAATTTGTATTATGTGATATTAACTAAAAAGTTAT
ACACAATAAAATTAACAAATGAATTATAAAAAATTAGACTGAAATGTCAAAAAAATTATAATATGATAAATTTTAGAAGAATGAGAA
AGTTTTGGGTGAAAAAAGCATAGGAAATCAGATAGAAAACCCAAGAGCTAATCACATAGAATTGTCCATAAAGAAAAAAAAAA
AAAACTCTATATATATCTATGCGATGATCCTGTTAATTCCACTGCATGTACACAAGCACCGCTCAAAAAAAAAAAAAAAAAAACA
GACATCTGATCTTAGTGATAAATATTATACACCTTACCACTAATG (998bp+81bp UTR = 1079bp)

Supplementary Figure S2. *Hevea PEP16* promoter sequence (NCBI GenBank accession number: MN200192).

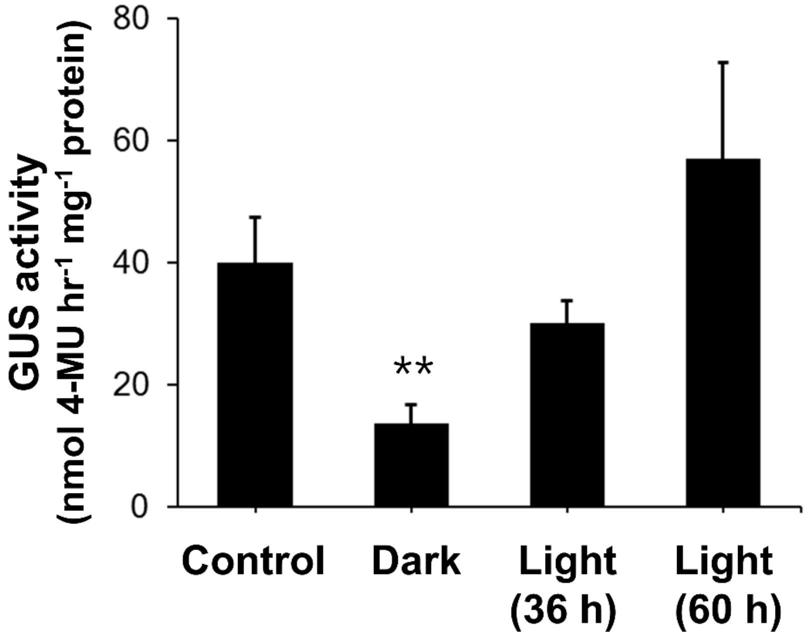

Supplementary Figure S3. Fluorometric GUS activity assay of transgenic *T. kok-saghyz* plants in response to external signals.

The effect of external signals such as non-treated control, dark stress and 36 h or 60 h light treatment after dark stress on fluorometric GUS activities driven by the *HbPEP16* promoter in transgenic *T. kok-saghyz* plants carrying p*PEP16::GUS* construct, which were at the different ages with those used in Figure 5 experiments. The asterisks indicate statistically significant difference (treatments versus control) as determined by *t*-tests: ** $P < 0.01$.

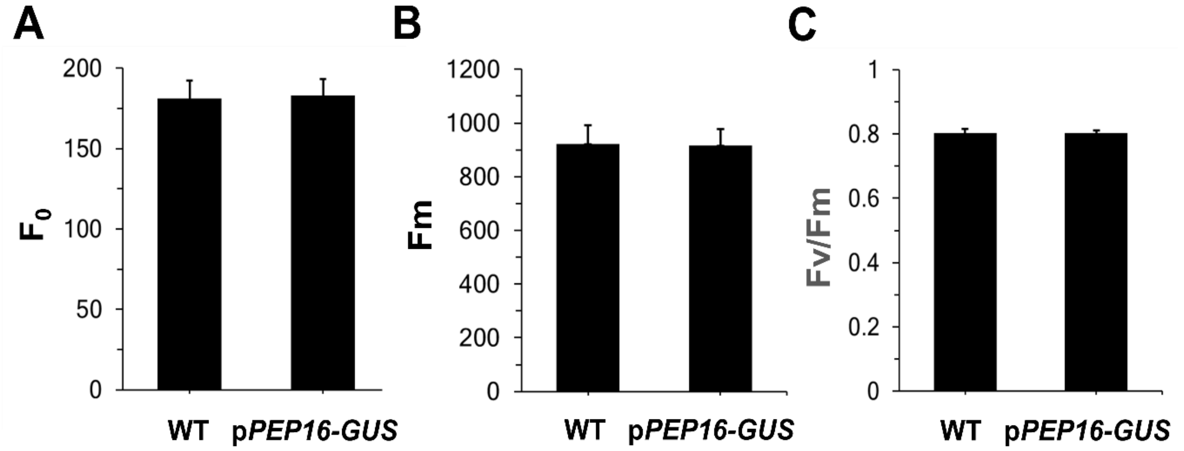

Supplementary Figure S4. The photosynthetic efficiency of *T. kok-saghyz* plants.

The chlorophyll fluorescence of WT and transgenic plants were measured; Fo, initial fluorescence intensity (A); Fm; maximum fluorescence intensity (B), Fv/Fm, maximum quantum yield of PSII (C). Each data represents the mean ± SD (n=12).
